# Supplementary material for: QuEChERS Extraction and Simultaneous Quantification in GC-MS/MS of Hexahydrocannabinol Epimers and Their Metabolites in Whole Blood, Urine, and Oral Fluid
Source: Molecules. 2024 Jul 22;29(14):3440. doi: 10.3390/molecules29143440 (PMC11279433; doi:10.3390/molecules29143440)
Supplement: Supplementary file 1 [file molecules-29-03440-s001.zip › molecules-3080021-supplementary.pdf]

Supplementary TableS1. Spiked calibrators, quality control samples and related working standard solutions concentrations for all the target analytes in the three different studied matrices

Matrix	Analyte	Cal 1 (ng mL^{-1})	WSTD1 (mg mL^{-1})	Cal 2 (ng mL^{-1})	WSTD2 (mg mL^{-1})	Cal 3 (ng mL^{-1})	WSTD3 (mg mL^{-1})	Cal 4 (ng mL^{-1})	WSTD4 (mg mL^{-1})	Cal 5 (ng mL^{-1})	WSTD5 (mg mL^{-1})	lQC (ng mL^{-1})	WSTD lQC (mg mL^{-1})	mQC (ng mL^{-1})	WSTD mQC (mg mL^{-1})	hQC (ng mL^{-1})	WSTD hQC (mg mL^{-1})
Blood	9(S)-HHC	1	0.02	2	0.04	5	0.1	15	0.3	30	0.6	3	0.06	12	0.24	24	0.48
	9(R)-HHC	1	0.02	2	0.04	5	0.1	15	0.3	30	0.6	3	0.06	12	0.24	24	0.48
	9aOH-HHC	1	0.02	2	0.04	5	0.1	15	0.3	30	0.6	3	0.06	12	0.24	24	0.48
	9bOH-HHC	1	0.02	2	0.04	5	0.1	15	0.3	30	0.6	3	0.06	12	0.24	24	0.48
	8(S)-HHC	1	0.02	2	0.04	5	0.1	15	0.3	30	0.6	3	0.06	12	0.24	24	0.48
	8(R)-HHC	1	0.02	5	0.1	10	0.2	30	0.6	50	1	3	0.06	20	0.4	40	0.8
	11OH-9(R)-HHC	1	0.02	2	0.04	5	0.1	15	0.3	30	0.6	3	0.06	12	0.24	24	0.48
	11OH-9(S)-HHC	1	0.02	2	0.04	5	0.1	15	0.3	30	0.6	3	0.06	12	0.24	24	0.48
	11nor-9(R)COOH	1	0.02	2	0.04	5	0.1	15	0.3	30	0.6	3	0.06	12	0.24	24	0.48
	11nor-9(S)COOH	1	0.02	5	0.1	10	0.2	30	0.6	50	1	3	0.06	20	0.4	40	0.8
Urine	9(S)-HHC	1	0.02	5	0.1	10	0.2	50	1	150	3	3	0.06	40	0.8	120	2.4

	9(R)-HHC	1	0.02	5	0.1	10	0.2	50	1	150	3	3	0.06	40	0.8	120	2.4
	9aOH-HHC	1	0.02	5	0.1	10	0.2	50	1	150	3	3	0.06	40	0.8	120	2.4
	9bOH-HHC	1	0.02	5	0.1	10	0.2	50	1	150	3	3	0.06	40	0.8	120	2.4
	8(S)-HHC	1	0.02	5	0.1	10	0.2	30	0.6	50	1	3	0.06	20	0.4	40	0.8
	8(R)-HHC	1	0.02	5	0.1	10	0.2	50	1	150	3	3	0.06	40	0.8	120	2.4
	11OH-9(R)-HHC	1	0.02	5	0.1	10	0.2	50	1	150	3	3	0.06	40	0.8	120	2.4
	11OH-9(S)-HHC	1	0.02	5	0.1	10	0.2	50	1	120	2.4	3	0.06	40	0.8	80	1.6
	11nor-9(R)COOH	1	0.02	5	0.1	10	0.2	30	0.6	50	1	3	0.06	20	0.4	40	0.8
	11nor-9(S)COOH	1	0.02	5	0.1	10	0.2	50	1	120	2.4	3	0.06	40	0.8	80	1.6
OF	9(S)-HHC	1	0.02	5	0.1	10	0.2	50	1	150	3	3	0.06	40	0.8	120	2.4
	9(R)-HHC	1	0.02	2	0.04	5	0.1	15	0.3	30	0.6	3	0.06	12	0.24	24	0.48
	9aOH-HHC	1	0.02	2	0.04	5	0.1	15	0.3	30	0.6	3	0.06	12	0.24	24	0.48
	9bOH-HHC	1	0.02	2	0.04	5	0.1	15	0.3	30	0.6	3	0.06	12	0.24	24	0.48
	8(S)-HHC	1	0.02	2	0.04	5	0.1	15	0.3	30	0.6	3	0.06	12	0.24	24	0.48
	8(R)-HHC	1	0.02	2	0.04	5	0.1	15	0.3	30	0.6	3	0.06	12	0.24	24	0.48
	11OH-9(R)-HHC	1	0.02	2	0.04	5	0.1	15	0.3	30	0.6	3	0.06	12	0.24	24	0.48

	11OH-9(S)-HHC	1	0.02	2	0.04	5	0.1	15	0.3	30	0.6	3	0.06	12	0.24	24	0.48
	11nor-9(R)COOH	1	0.02	2	0.04	5	0.1	15	0.3	30	0.6	3	0.06	12	0.24	24	0.48
	11nor-9(S)COOH	1	0.02	2	0.04	5	0.1	15	0.3	30	0.6	3	0.06	12	0.24	24	0.48
